# Improving Nearest Relative Legal Literacy and Support Under the Mental Health Act: A Quality Improvement Project in an Autism-Informed Inpatient Service

**DOI:** 10.1192/bjo.2026.11386

**Published:** 2026-06-30

**Authors:** Afifa Ghouri, Azmathulla Khan Hameed

**Affiliations:** Cygnet Hospital Harrow, London, United Kingdom

## Abstract

**Aims:**

This quality improvement project aimed to assess and improve NR legal understanding, confidence, and perceived emotional support within Springs Services. The primary objective was to increase the proportion of NRs who felt adequately informed about their role under the MHA to at least 80%.

**Methods:**

Nearest Relatives (NRs) have a statutory safeguarding role under the Mental Health Act (MHA) 1983; however, existing evidence indicates that many feel inadequately informed about their legal rights and responsibilities. Prior research describes inconsistent explanations and assumptions of prior knowledge, contributing to uncertainty and distress during compulsory admissions. These challenges may be particularly pronounced in autism-informed inpatient services, where families are often navigating complex clinical and legal processes simultaneously.

A structured baseline survey was distributed to NRs of patients admitted to Springs Services. The questionnaire included Likert-scale items assessing understanding of the NR role, clarity of MHA explanations, involvement in care and discharge planning, confidence in raising concerns, and emotional support, alongside yes/no questions and free-text comments. Twenty completed responses were analysed descriptively. Qualitative feedback was reviewed thematically to identify priority areas for improvement. Informed by baseline findings, service-level interventions were introduced, including an autism-informed NR information leaflet, a standardised admission explanation script, a brief NR support check-in, and focused staff guidance on trauma and autism informed communication.

**Results:**

Baseline data demonstrated variable NR experiences. While approximately 60% of respondents agreed or strongly agreed that they understood their role as an NR, fewer (around 45%) reported confidence in their legal rights or a clear understanding of the distinction between Nearest Relative and Next of Kin. Only around 50% recalled receiving written information about their role at the point of admission. Communication from the ward was rated positively by approximately two-thirds of respondents; however, lower scores were reported for emotional support (around 40%) and involvement in discharge planning(approximately 35%). Confidence in speaking up or asking questions was reported by just over half of respondents. Free-text comments highlighted confusion about legal processes, uncertainty about who to contact for advice, and a desire for earlier, clearer explanations delivered in accessible language.

**Conclusion:**

This project identified measurable gaps in NR legal literacy, confidence, and emotional support within an autism-informed inpatient service. Baseline findings support the need for structured, accessible information and proactive engagement with NRs to strengthen safeguards under the MHA. Ongoing data collection will evaluate whether these targeted interventions increase the proportion of NRs who feel adequately informed and supported, with the aim of embedding sustainable improvements in statutory practice and family involvement.

